# Syringaldehyde inhibits NFκB-mediated inflammation and apoptosis and activates Nrf2/HO-1 signaling to reduce HCl/ethanol-induced gastric injury

**DOI:** 10.1007/s00210-026-05490-8

**Published:** 2026-05-26

**Authors:** Berrin Yalinbas-Kaya, Selcan Cesur, Sinan Ince

**Affiliations:** 1 Department of Gastroenterology, University of Health Sciences, Ministry of Health Eskisehir City Hospital, 26080 Eskisehir, Türkiye; 2 Faculty of Veterinary Medicine, Department of Pharmacology and Toxicology, University of Afyon Kocatepe, 03200 Afyonkarahisar, Turkey

**Keywords:** Syringaldehyde, Gastric ulcer, Anti-inflammatory effect, Nrf2/HO-1, Apoptosis, Oxidative stress

## Abstract

Syringaldehyde (SG) is a naturally derived phenolic aldehyde known for a range of biological activities. This study evaluated its protective potential against the chemically induced gastric injury model and investigated the underlying mechanisms involved. Acute gastric ulceration was induced in male Swiss albino mice by intragastric administration of a mixture of 0.3 M HCl and 70% ethanol. SG was orally administered at 25 and 50 mg/kg, and omeprazole (20 mg/kg) served as the reference anti-ulcer drug. HCl/ethanol exposure caused severe gastric mucosal injury characterized by increased lipid peroxidation, elevated levels of inflammatory mediators (IL-6, NFκB, iNOS, Cox-2, TNF-α, and IL-1β), and enhanced pro-apoptotic markers Bax and Cas-3. In parallel, antioxidant defense mechanisms were markedly impaired, as indicated by decreased GSH levels, reduced SOD and CAT activities, diminished mucosal protective mediators (PGE2 and NO), and downregulation of HO-1, Nrf2, and Bcl-2 expression. Treatment with SG significantly attenuated oxidative stress, gastric mucosal damage, and inflammatory responses, and modulated apoptosis-related markers. These protective effects were accompanied by restoration of antioxidant enzyme activities and increased levels of PGE2 and NO, together with upregulation of Nrf2/HO-1 expression. Overall, these results suggest that SG may exert gastroprotective effects against gastric injury through modulation of oxidative stress, inflammation, and apoptotic pathways.

## Introduction

Ulcerative lesions developing in the gastric mucosa constitute a significant component of stomach-related disorders (Cho et al. 2023). Excessive alcohol intake, tobacco use, chronic stress exposure, uncontrolled consumption of NSAIDs, and unhealthy nutritional patterns constitute the principal contributing factors in the development of gastric ulcer disease, which is associated with a substantial potential for malignant progression (Ford et al. 2016; Liu et al. 2021). Inflammation and oxidative stress are considered the main factors in the development of gastric ulcers, and anti-inflammatory and antioxidant-based strategies are widely investigated for ulcer prevention (Yu et al. 2020). Furthermore, gastric acid secretion exacerbates the acidic environment, thereby contributing to the progression of gastric mucosal injury (Boby et al. 2021). Therefore, the gastric ulcer model employed in this study is frequently used to evaluate agents with potential inhibitory effects on ulcer pathogenesis and progression (Liu et al. 2021).

Nrf2 functions as a key transcriptional regulator that maintains cellular balance under conditions of oxidative challenge (Tureyen et al. 2023). Under stress conditions, Nrf2 binds to antioxidant response elements, promoting the expression of cytoprotective genes (Cesur et al. 2025). Nrf2/HO-1 activation limits oxidative damage and restrains inflammation by inhibiting NFκB and lowering TNF-α production (Yalinbas-Kaya et al. 2025). Additionally, Cox-2 is closely associated with the NFκB signaling pathway and may play a role in NFκB-mediated inflammatory responses (Hassanin et al. 2022). Furthermore, increased NFκB activity induces apoptosis through caspase activation (Alasmari et al. 2021). Accordingly, mitigating inflammation-driven processes primarily involves enhancing Nrf2 activity while restraining NFκB signaling, which limits pro-inflammatory cytokine production and ultimately reduces apoptotic events (Ince et al. 2024).

Conventional anti-ulcer pharmacotherapy is frequently limited by undesirable side effects, which has led to increasing scientific interest in biologically active compounds of natural origin (Boby et al. 2021; Serafim et al. 2021).

Current experimental findings suggest that these molecules do not act through a single dominant mechanism; instead, they influence multiple, interrelated cellular processes. Naturally derived compounds have been associated with regulation of redox balance, suppression of inflammatory signaling, and modulation of apoptosis, all of which contribute to tissue protection (Nelson et al. 2022; Awad et al. 2023; Hamza et al. 2023). Evidence from diverse experimental models indicates that these effects are mediated through coordinated regulation of intracellular defense systems and pro-inflammatory cascades, ultimately contributing to the stabilization of cellular function and structural integrity (Othman et al. 2023; Botros et al. 2024). Such multi-layered biological activity supports the idea that these compounds may provide a generalized protective framework that is not restricted to a specific organ system but may also be relevant to the pathophysiology of gastric mucosal injury (Nelson et al. 2022; Othman et al. 2023).

Within this mechanistic framework, syringaldehyde (SG) emerges as a promising candidate compound. This low–molecular-weight phenolic aldehyde, derived from lignin and identified in plant sources such as Magnolia officinalis and Manihot esculenta, has attracted attention due to its diverse biological effects (Wen et al. 2024). Previous experimental observations have shown that SG is capable of limiting the proliferation of various pathogenic microorganisms, including *Klebsiella pneumoniae*, *Pseudomonas aeruginosa*, and *Staphylococcus aureus* (Fillat et al. 2012). In addition to its antimicrobial activity, SG has been associated with reduced cellular proliferation in malignant models. It has also been reported to alleviate tissue damage under conditions such as ischemic injury and chemically induced cardiotoxicity. These effects have been linked to its ability to influence oxidative status and apoptotic processes (González-Sarrías et al. 2012; Bozkurt et al. 2014; Shahzad et al. 2018). Moreover, experimental data indicating its involvement in glycemic regulation further point to a broader physiological relevance (Huang et al. 2012).

Despite the accumulation of evidence supporting these diverse biological actions, the potential contribution of SG to gastric mucosal protection remains insufficiently explored. Accordingly, the present study was designed to evaluate its effects in an experimental model of acute gastric injury, using a comprehensive approach that integrates biochemical measurements, molecular analyses, and histopathological evaluation. Given that gastric ulcer pathogenesis is closely associated with inflammation, oxidative stress, and apoptosis, and considering the previously reported regulatory effects of SG on these pathways, it is reasonable to hypothesize that SG may exert protective effects in gastric mucosal injury.

## Materials and methods

### Experimental reagents

Omeprazole used in the study was obtained from Terra Pharmaceutical Industry (Istanbul, Türkiye). SG, ethanol, HCl, and DMSO were purchased from Sigma-Aldrich (Missouri, USA). All additional analytical grade reagents were obtained from reputable commercial sources.

### Study design

The animals were supplied by the Afyon Kocatepe Experimental Animal Research Center. All procedures involving animals were carried out in compliance with internationally recognized ethical standards and were approved by the Local Ethics Committee (49,533,702/342). The mice were kept under controlled environmental conditions (12-h light/dark cycle, 55–60% relative humidity, and 22 ± 2 °C). During the experimental period, animals had unrestricted access to standard laboratory chow and drinking water. Before induction of gastric ulcer, food was withdrawn for 24 h, while water was provided ad libitum*.*

Forty-nine male Swiss albino mice (4–5 months old, 30–40 g) were utilized in this experiment and randomly assigned to seven groups, each comprising seven animals. The doses of HCl/ethanol (Zhang et al. 2021), SG (Tureyen et al. 2025), and omeprazole (Tureyen and Ince 2021) used in the experimental phase were determined based on previously published studies.

The SG doses (25 and 50 mg/kg) were selected based on previously published in vivo studies demonstrating its antioxidant and anti-inflammatory properties together with a favorable safety profile. In the present experiment, no signs of toxicity or mortality were detected in SG-treated animals. According to body surface area–based dose conversion, these doses correspond to approximately 2–4 mg/kg in humans, indicating potential translational relevance. Omeprazole (20 mg/kg) was included as a reference anti-ulcer agent due to its well-established role as a proton pump inhibitor in both experimental models and clinical management of gastric mucosal injury. It was administered as a separate treatment group, and no co-administration with SG was performed. Details of the experimental design are listed in Table 1.

**Table 1:** Design of experimental groups and treatments

Group	Treatment	Dose/Administration Route	Purpose/Description
Control	Physiological saline	0.1 cc/10 g, p.o	Negative control group: received no drug treatment
DMSO	0.1% DMSO	0.1 cc/10 g, p.o	Solvent control group to assess possible DMSO effects
SG_50_	Syringaldehyde	50 mg/kg, p.o	Received syringaldehyde alone to assess its baseline effect
HCl/EtOH	0.3 M HCl + 70% EtOH	0.1 cc/10 g, p.o	Ulcer-induced group to create gastric mucosal injury
SG_25_ + HCl/EtOH	Syringaldehyde + HCl/EtOH	25 mg/kg Syringaldehyde (p.o.) + 0.1 cc/10 g HCl/EtOH (p.o.)	To evaluate the gastroprotective effect of syringaldehyde
SG_50_ + HCl/EtOH	Syringaldehyde + HCl/EtOH	50 mg/kg Syringaldehyde (p.o.) + 0.1 cc/10 g HCl/EtOH (p.o.)	To evaluate the gastroprotective effect of syringaldehyde
Omeprazole + HCl/EtOH	Omeprazole + HCl/EtOH	20 mg/kg Omeprazole (p.o.) + 0.1 cc/10 g HCl/EtOH (p.o.)	Reference (positive control) group: standard anti-ulcer drug

SG and omeprazole were prepared in 0.1% DMSO and administered orally by gavage once daily for three days. This concentration of DMSO is considered biologically inactive, and accordingly, no significant differences were detected between the control and vehicle-treated groups, indicating no interference of the solvent with the experimental results. One hour after the final administration, gastric ulceration was induced using HCl/ethanol.

At the end of the 4-h post-induction period, animals were anesthetized with ketamine (80 mg/kg, i.p.) and xylazine (10 mg/kg, i.p.). Blood samples were collected via intracardiac puncture, and euthanasia was subsequently performed under deep anesthesia in accordance with ethical standards. The stomach tissues were then removed for further analyses. Serum was obtained by centrifuging blood samples (600 × g −15 min). Gastric tissues were homogenized in Tris–HCl buffer (pH 7.4; 0.15 M) and centrifuged (2,500 rpm-10 min). The resulting supernatants and serum samples were stored at −80 °C until further analysis.

### Determination of gastric pH and gastric wall mucus

Prior to gastric tissue collection, the pylorus was ligated, and the gastric contents were subsequently collected and centrifuged (4.000 rpm-15 min). Gastric juice pH was determined using a pH meter (Sartorius, Germany) (Zhou et al. 2020).

Gastric mucus content was evaluated using the Alcian blue binding method. A standardized portion of gastric tissue was immersed in Alcian blue solution (10 mL; 0.02%) prepared in a buffer (sucrose-0.16 M and sodium acetate 0.05 M; pH 5.8) and incubated (25 °C—24 h) to facilitate dye binding. After incubation, the samples underwent centrifugation (3,000 rpm; 10 min; 4 °C). The supernatant’s absorbance was recorded at 620 nm with a spectrophotometer. Mucus content was expressed as mg per g of tissue and quantified based on Alcian blue binding, with concentrations calculated from a standard calibration curve generated using known dye solutions (Ribeiro et al. 2016).

### Macroscopic examination of gastric tissue and ulcer lesions

Following removal, the stomachs were incised longitudinally along the greater curvature, gently washed with isotonic saline, and subjected to gross morphological inspection. The extent of gastric injury was assessed independently by observers blinded to the experimental groups. Lesions were graded according to a standardized scoring system: 0 (absence of lesions), 1–2 (minor mucosal damage), 3–4 (localized ulcerative lesions), 5–6 (pronounced ulcer formation), and 7 (severe and widespread ulceration) (Byeon et al. 2018). In addition, photographic documentation of the gastric mucosa was obtained, and ulcerated regions were quantitatively evaluated using digital image analysis software (ImageJ, version 1.53r; NIH, USA). The total damaged mucosal surface was calculated and expressed in square millimeters (Yoo et al. 2022).

### Biochemical analyses

Levels of malondialdehyde (MDA) and GSH were measured according to the methods of Ohkawa et al. (1979) and Draper and Hadley (1990). Superoxide dismutase (SOD), catalase (CAT), and total protein were measured using Sinha (1972), Sun et al. (1988), and (Lowry et al. 1951), respectively. All absorbance measurements were performed with a spectrophotometer (Shimadzu Corp., Tokyo, Japan). Furthermore, levels of IL-6 (Cat. No. SL0326Mo), PGE2 (Cat. No. SL0461Mo), and NO (Cat. No. SL0615Mo) in serum and gastric tissue samples were determined by ELISA using commercially available kits obtained from Sunlong Biotech (Zhejiang, China).

### mRNA gene expression analysis

Total RNA was extracted from gastric tissue specimens using the A.B.T.™ Blood/Tissue RNA Purification Kit. The isolated RNA was subsequently reverse-transcribed into complementary DNA (cDNA). Gene expression analysis at the mRNA level was carried out by quantitative PCR, targeting genes associated with inflammation, apoptosis, and antioxidant defense, including NFκB, Cox-2, iNOS, IL-1β, TNF-α, Bax, Nrf2, Bcl-2, HO-1, and Cas-3. Custom-designed oligonucleotide primers were obtained from Sentegen Biotechnology (Ankara, Türkiye), and the corresponding sequences are presented in Table 2. All reactions were performed in triplicate to ensure reproducibility. Normalized expression ratios were derived via the 2⁻ΔΔCt calculation, using β-actin as the internal standard (Tureyen and Ince 2021).

**Table 2:** Description of polymerase chain reaction primers (β-actin, NFκB, Cox-2, iNOS, TNF-α, IL1-β, Cas-3, Bax, Bcl-2, Nrf2, and HO-1), sequence, and primer length

Gene	Primer Sequence (5’–3’)	Primer Length (bp)
β-actin	F: AAGGCCAACCGTGAAAAGAT R: GTGGTACGACCAGAGGCATAC	20/21
NFκB	F: AGGAAGGCAAAGCGAATCCA R: TCAGAACCAAGAAGGACGGC	20/20
Cox-2	F: TTGCTCTCCCCTCTCTACGC R: GCAGTCGTAGTTCACCAGGTT	20/21
iNOS	F: AACTTGTTTGCAGGCGTCAG R: CACATTGCTCAGGGATGGA	20/19
TNF-α	F: ATCCATCTCTTTGCGGAGGC R: GGGGGAGAGGTAGGGATGTT	20/20
IL-1β	F: TGCCACCTTTTGACAGTGATG R: TGATGTGCTGCTGCGAGATT	21/20
Cas-3	F: AGCTTGGAACGGTACGCTAA R: CCACTGACTTGCTCCCATGT	20/20
Bax	F: TGCTAGCAAACTGGTGCTCA R: ATGTGGGGGTCCCGAAGTA	20/19
Bcl-2	F: GAACTGGGGGAGGATTGTTGG R: GCATGCTGGGGCCATATAGT	20/20
Nrf2	F: CATGTGTGGCAGTCCATGATTT R: AGTACTGTACAGCAGGCATAC	22/21
HO-1	F: CCTCACAGATGGCGTCACTT R: TGGGGGCCAGTATTGCATTT	20/20

### Histopathological analysis

After fixation in 10% neutral buffered formalin, gastric specimens were processed and embedded in paraffin. Thin Sects. (5 μm) were then obtained from the blocks using a Leica microtome (Nussloch, Germany) (Tureyen et al. 2023). H&E staining was applied to examine morphological changes in the gastric mucosal architecture. Observations were made using light microscopy, and images were recorded with a Nikon DS-Fi3 digital imaging system (Tokyo, Japan).

### Immunohistochemical evaluation

For immunohistochemical analysis, paraffin sections were deparaffinized, rehydrated, and treated to block endogenous peroxidase activity using 1% H₂O₂. Antigen retrieval was performed by heat exposure in citrate buffer. After washing, sections were incubated with primary antibodies (Table 3) at 4 °C overnight, followed by HRP-labeled secondary antibody incubation. Immunostaining was visualized with DAB, counterstained with hematoxylin, and evaluated under light microscopy (200 ×).

**Table 3:** Primary antibodies, dilutions, and catalog numbers used in immunohistochemical analyses

Antibodies	Dilutions	Catalog number
Bax	1:100	E-AB-13814
Bcl-2	1:100	E-AB-60012
NFκB	1:200	E-AB-60843
Nrf2	1:200	E-AB-93081
Cox-2	1:200	E-AB-68287
HO-1	1:200	E-AB-66079
iNOS	1:250	E-AB-70051
Cas-3	1:100	E-AB-30004
IL-1β	1:100	PAA563Ra01
TNF-α	1:100	PAA133Ra01

### Data evaluation approach

All datasets were analyzed using GraphPad Prism (v8.0). Distribution characteristics were assessed prior to statistical testing. Differences among groups were analyzed using one-way ANOVA with Tukey’s post hoc test. Data are reported as mean ± standard deviation, and a p-value below 0.05 was considered statistically significant.

## Results

### Protective effect of SG against HCl/ethanol-induced gastric ulcer

**Fig. 1:**
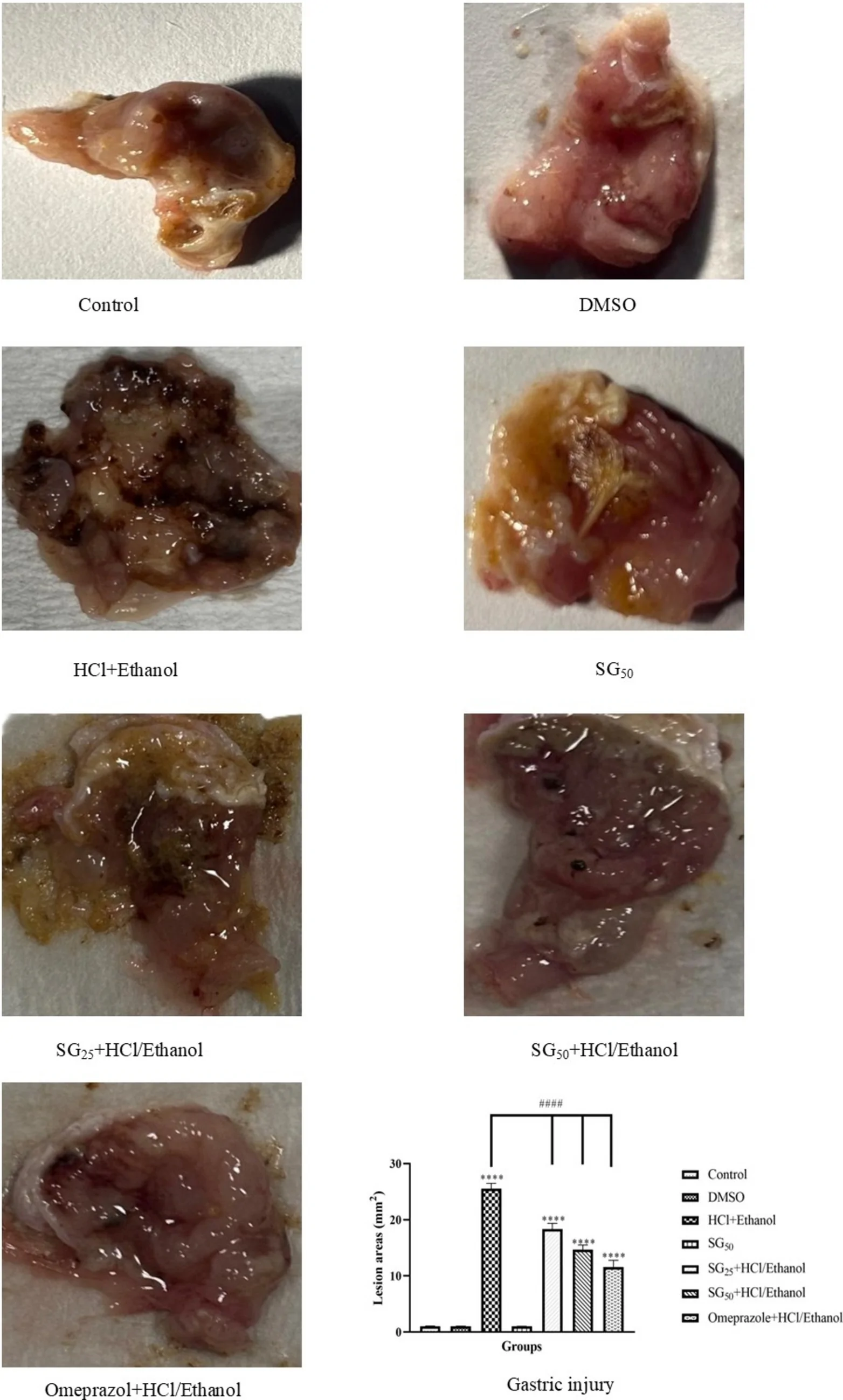
Macroscopic appearance of gastric mucosa and gastric injury index in ethanol/HCl-induced gastric ulcer model in mice. Representative macroscopic images of gastric tissues from Control, DMSO, HCl/Ethanol, SG₅₀, SG₂₅ + HCl/Ethanol, SG₅₀ + HCl/Ethanol, and Omeprazole + HCl/Ethanol groups are shown. Ethanol/HCl administration induced severe gastric mucosal hemorrhage and ulcerative lesions, whereas pretreatment with syringaldehyde (SG) at doses of 25 and 50 mg/kg markedly attenuated gastric damage in a dose-dependent manner, comparable to omeprazole. The bar graph represents the gastric injury index (lesion area, mm^2^). Data are expressed as mean ± SD. ****p < 0.0001 vs. Control; ####*p* < 0.001 vs. HCl/Ethanol group

Macroscopic examination revealed no detectable alterations in the gastric mucosa of mice in the control, DMSO, and SG (50 mg/kg) groups. In contrast, mice subjected to HCl/ethanol administration exhibited extensive hemorrhagic ulcerative lesions throughout the gastric mucosa, characterized by prominent hemorrhagic areas and severe mucosal injury (Fig. 1). Compared with the alcohol-treated group, SG treatment (25 and 50 mg/kg) in a dose-dependent manner significantly reduced the formation of HCl/ethanol-induced gastric ulcers (*p* < 0.0001). Similarly, omeprazole administration markedly attenuated HCl/ethanol-induced gastric damage. Quantitative analysis demonstrated that the ulcerated mucosal area in the HCl/ethanol group increased by 75.37% relative to the normal control group (*p* < 0.0001). In contrast, SG (25 and 50 mg/kg) and omeprazole significantly decreased gastric mucosal damage by 27.40%, 42.48%, and 54.62%, respectively, compared with the HCl/ethanol group (*p* < 0.0001).

**Fig. 2:**
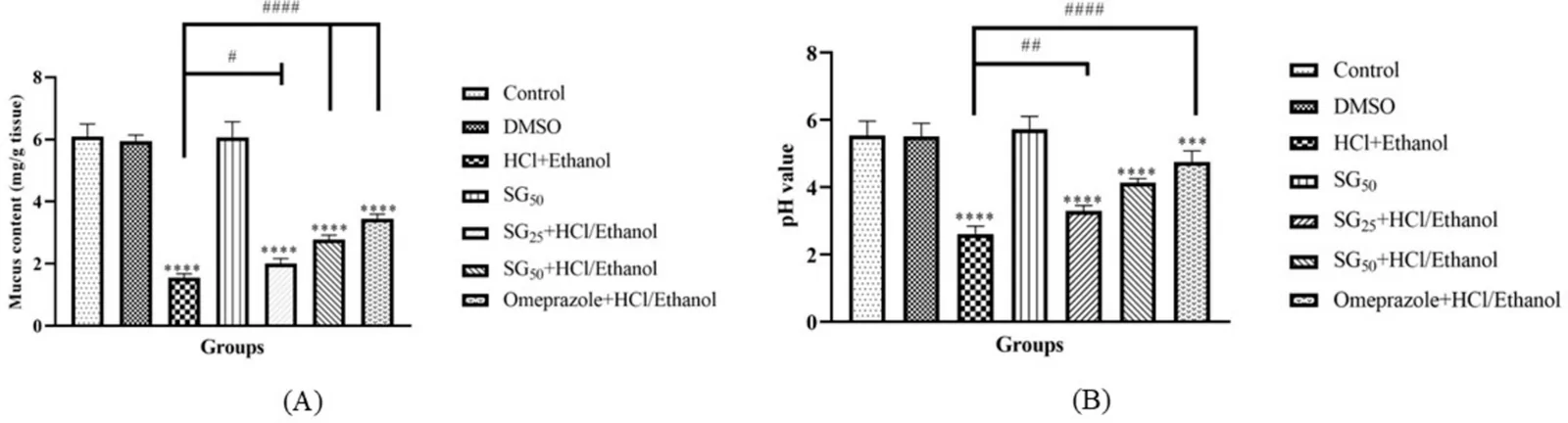
Effects of syringaldehyde (SG) on gastric mucus content and gastric pH in ethanol/HCl-induced gastric injury in mice. (**A**) Gastric mucus content (mg/g tissue). (**B**) Gastric pH values. Ethanol/HCl administration significantly reduced gastric mucus content and gastric pH compared with the control group. Pretreatment with syringaldehyde (SG) at doses of 25 and 50 mg/kg markedly restored mucus content and pH levels in a dose-dependent manner, with effects comparable to omeprazole. Data are presented as mean ± SD. ****p < 0.0001, ***p < 0.001 vs. Control; #*p* < 0.05, ##*p* < 0.01, ####*p* < 0.0001 vs. HCl/Ethanol group

Evaluation of gastric mucus secretion demonstrated a pronounced depletion in the alcohol-exposed group when compared with the untreated control animals (*p* < 0.0001). In contrast, SG (25 and 50 mg/kg), as well as omeprazole, resulted in a significant preservation of gastric mucus relative to the alcohol-treated group (*p* < 0.0001). Quantitative analysis showed that alcohol exposure led to a 74.52% reduction in mucus content compared with controls. Treatment with SG at 25 mg/kg partially restored mucus levels by 7.55% (*p* < 0.0457), whereas SG at 50 mg/kg and omeprazole produced more pronounced increases of 20.19% and 31.03%, respectively (both *p* < 0.0001), when compared with the alcohol group (Fig. 2A). No statistically meaningful changes were detected between the control group and animals receiving DMSO or SG alone at 50 mg/kg.

Gastric acidity was significantly increased following HCl/ethanol administration, reflected by a marked decrease in gastric pH compared with the control group (*p* < 0.0001). Treatment with SG significantly mitigated this effect. SG at 25 mg/kg moderately increased gastric pH values (*p* < 0.0032), whereas SG at 50 mg/kg and omeprazole produced a stronger restoration of gastric pH (both *p* < 0.0001) relative to the HCl/ethanol group (Fig. 2B). These findings indicate that SG confers protection against HCl/ethanol-induced gastric injury by improving gastric mucus integrity and contributing to the regulation of intragastric pH.

**Fig. 3:**
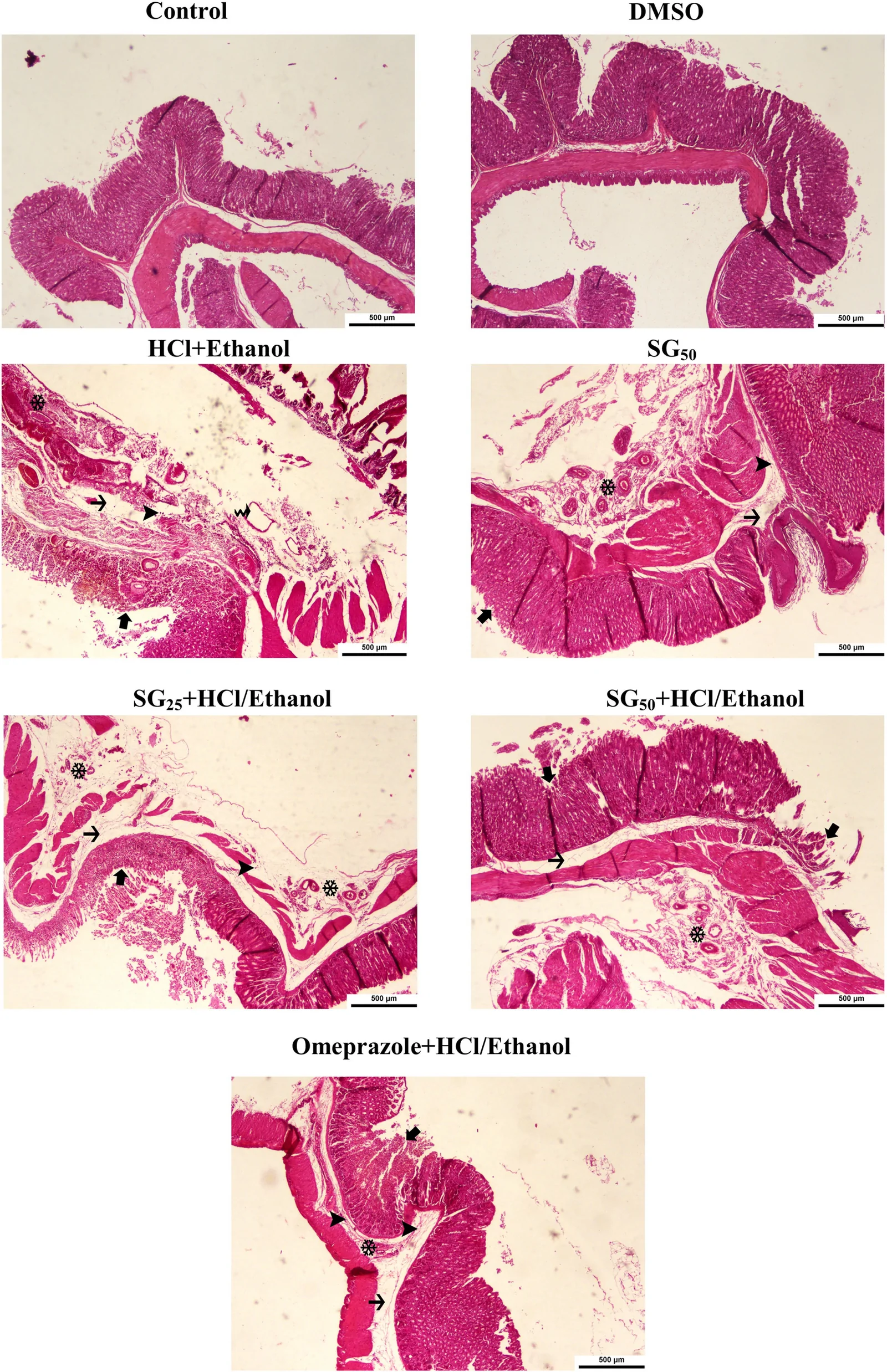
Histopathological evaluation of gastric tissue in ethanol/HCl-induced gastric ulcer model in mice (H&E staining). Representative hematoxylin and eosin–stained gastric tissue sections from Control, DMSO, HCl/Ethanol, SG₅₀, SG₂₅ + HCl/Ethanol, SG₅₀ + HCl/Ethanol, and Omeprazole + HCl/Ethanol groups are shown. The control and DMSO groups exhibited normal gastric mucosal architecture. In the HCl/Ethanol group, severe gastric injury was observed, characterized by desquamation and epithelial sloughing (thick arrows), edema in the submucosa and serosa (thin arrows), mononuclear cell infiltration in the submucosa and serosa (arrowheads), dilation of lymphatic vessels (curved arrows), and vascular hyperemia (asterisks). Pretreatment with syringaldehyde (SG) at doses of 25 and 50 mg/kg markedly attenuated these histopathological alterations, preserving mucosal integrity and reducing inflammatory infiltration in a dose-dependent manner. Omeprazole treatment also demonstrated pronounced gastroprotective effects. Original magnification: × 100

Histopathological evaluation of gastric tissues stained with hematoxylin and eosin (H&E) (Fig. 3) revealed no detectable pathological alterations in the control, DMSO, and SG (50 mg/kg) groups. In contrast, pronounced histological damage was observed in the HCl/ethanol-treated group, including epithelial desquamation and shedding, submucosal edema, submucosal mononuclear cell infiltration, lymphatic vessel dilation, and vasodilation (*p* < 0.0001) (Table 4). Notably, administration of SG (25 and 50 mg/kg) significantly attenuated these microscopic lesions compared with the alcohol group (*p* < 0.0001). Similarly, omeprazole treatment markedly reduced histopathological damage relative to the alcohol group, indicating a protective effect on the gastric mucosa.

**Table 4:** Histopathological alterations in gastric tissue following administration of syringaldehyde (SG) against HCl/EtOH exposure

Group	Desquamation and shedding	Submucosal edema	Submucosal mononuclear cell infiltration	Lymphatic vessel dilation	Vasodilation
Control	0.00 ± 0.00	0.00 ± 0.00	0.00 ± 0.00	0.00 ± 0.00	0.00 ± 0.00
DMSO	0.00 ± 0.00	0.00 ± 0.00	0.00 ± 0.00	0.00 ± 0.00	0.00 ± 0.00
HCl/EtOH	2.53 ± 0.06	1.93 ± 0.05	1.55 ± 0.06	2.14 ± 0.04	2.45 ± 0.07
SG_50_	0.00 ± 0.00	0.00 ± 0.00	0.00 ± 0.00	0.00 ± 0.00	0.00 ± 0.00
SG_25_ + HCl/EtOH	2.32 ± 0.04	1.70 ± 0.04	1.35 ± 0.04	1.90 ± 0.03	1.98 ± 0.08
SG_50_ + HCl/EtOH	2.20 ± 0.04	1.40 ± 0.04	1.11 ± 0.06	1.67 ± 0.05	1.57 ± 0.04
Omeprazole + HCl/EtOH	1.98 ± 0.06	1.22 ± 0.03	0.85 ± 0.05	1.63 ± 0.05	1.52 ± 0.05
*P* value	0.0001	0.0001	0.0001	0.0001	0.0001

Data are expressed as mean ± SD (*n* = 7). Statistical significance was determined by one-way ANOVA followed by Tukey’s post hoc test (*p* < 0.05)

### Protective effects of SG on oxidative stress–associated biochemical markers in the gastric tissue of HCl/ethanol-exposed mice

**Fig. 4:**
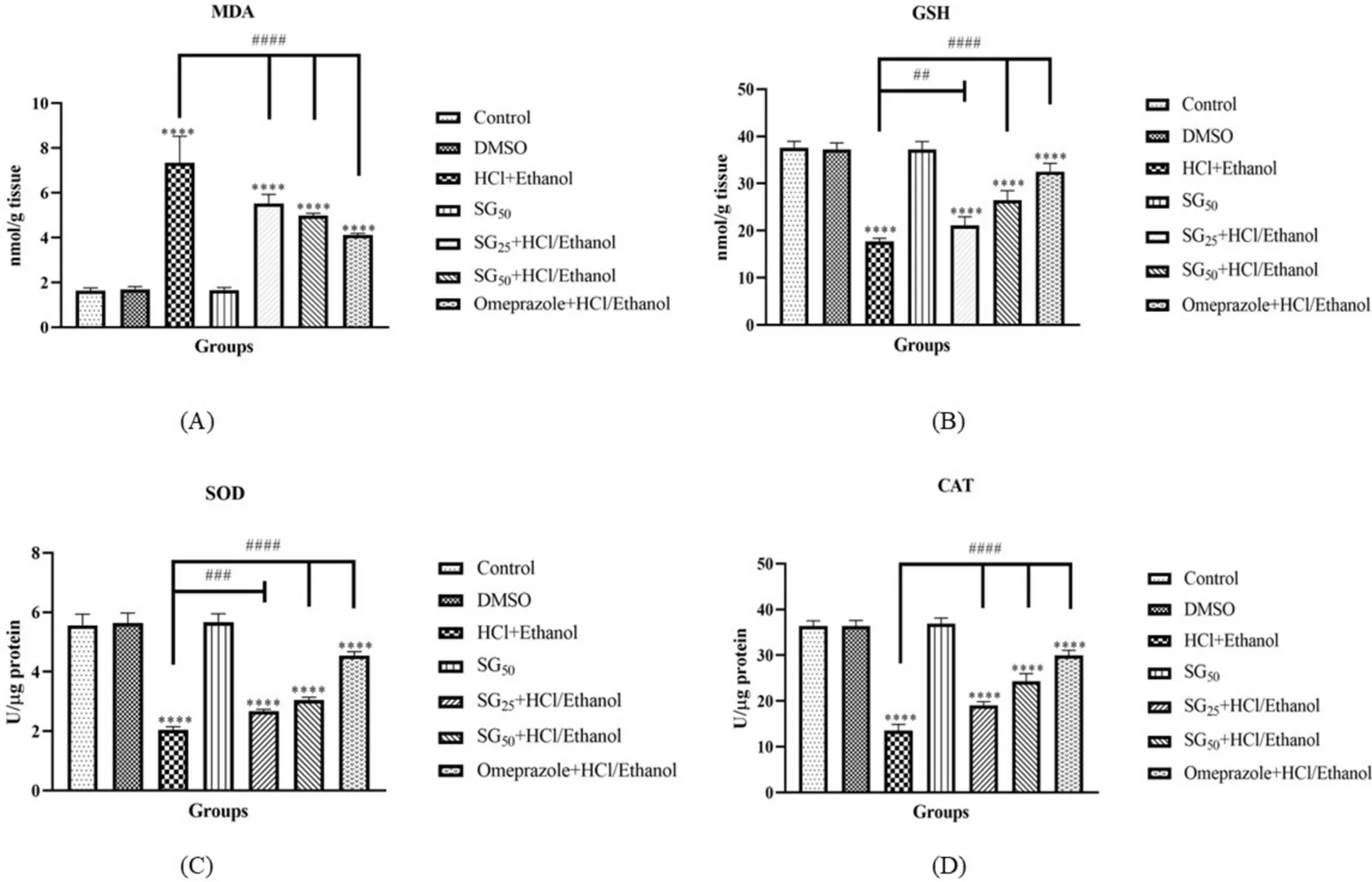
Effects of syringaldehyde (SG) on oxidative stress and antioxidant defense parameters in gastric tissue of ethanol/HCl-induced gastric injury in mice. (**A**) Malondialdehyde (MDA) levels, (**B**) reduced glutathione (GSH) content, (**C**) superoxide dismutase (SOD) activity, and (**D**) catalase (CAT) activity in gastric tissue homogenates. Ethanol/HCl administration significantly increased MDA levels while markedly decreasing GSH content and SOD and CAT activities compared with the control group, indicating severe oxidative stress. Pretreatment with syringaldehyde (SG) at doses of 25 and 50 mg/kg significantly attenuated lipid peroxidation and restored antioxidant parameters in a dose-dependent manner, with effects comparable to omeprazole. Data are expressed as mean ± SD. ****p < 0.0001 vs. Control; ##*p* < 0.01, ###*p* < 0.001, ####*p* < 0.0001 vs. HCl/Ethanol group

Given that gastric damage induced by HCl/ethanol is closely linked to oxidative stress and disruption of endogenous antioxidant mechanisms, indices of lipid peroxidation and antioxidant status—including MDA, GSH, and the activities of SOD and CAT—were analyzed in gastric tissue homogenates. Exposure to HCl/ethanol caused a pronounced elevation in gastric MDA levels (Fig. 4A), indicating enhanced lipid peroxidation. This increase was significantly mitigated by treatment with SG at 25 and 50 mg/kg, as well as by omeprazole at 20 mg/kg (*p* < 0.0001).

Exposure to HCl/ethanol resulted in a significant depletion of gastric GSH levels, whereas this reduction was markedly alleviated by SG administration at 25 mg/kg (*p* < 0.0042), SG at 50 mg/kg (*p* < 0.0001), and omeprazole at 20 mg/kg (*p* < 0.0001) (Fig. 4B). Similarly, the suppressed activities of key antioxidant enzymes were restored following these treatments. SOD activity showed a significant increase (Fig. 4C; *p* < 0.0002, *p* < 0.0001, and *p* < 0.0001, respectively), and CAT activity was also significantly elevated (Fig. 4D; *p* < 0.0001) compared with the HCl/ethanol group. No notable alterations in these parameters were detected in the DMSO group or in animals receiving SG alone (50 mg/kg) when compared with controls.

Overall, these findings indicate that SG exerts protective effects against HCl/ethanol-induced gastric injury by reducing oxidative damage and enhancing the antioxidant defense capacity of gastric tissue.

### Modulatory effects of SG on inflammation-associated markers in the serum and gastric tissue of HCl/ethanol-exposed mice

**Fig. 5:**
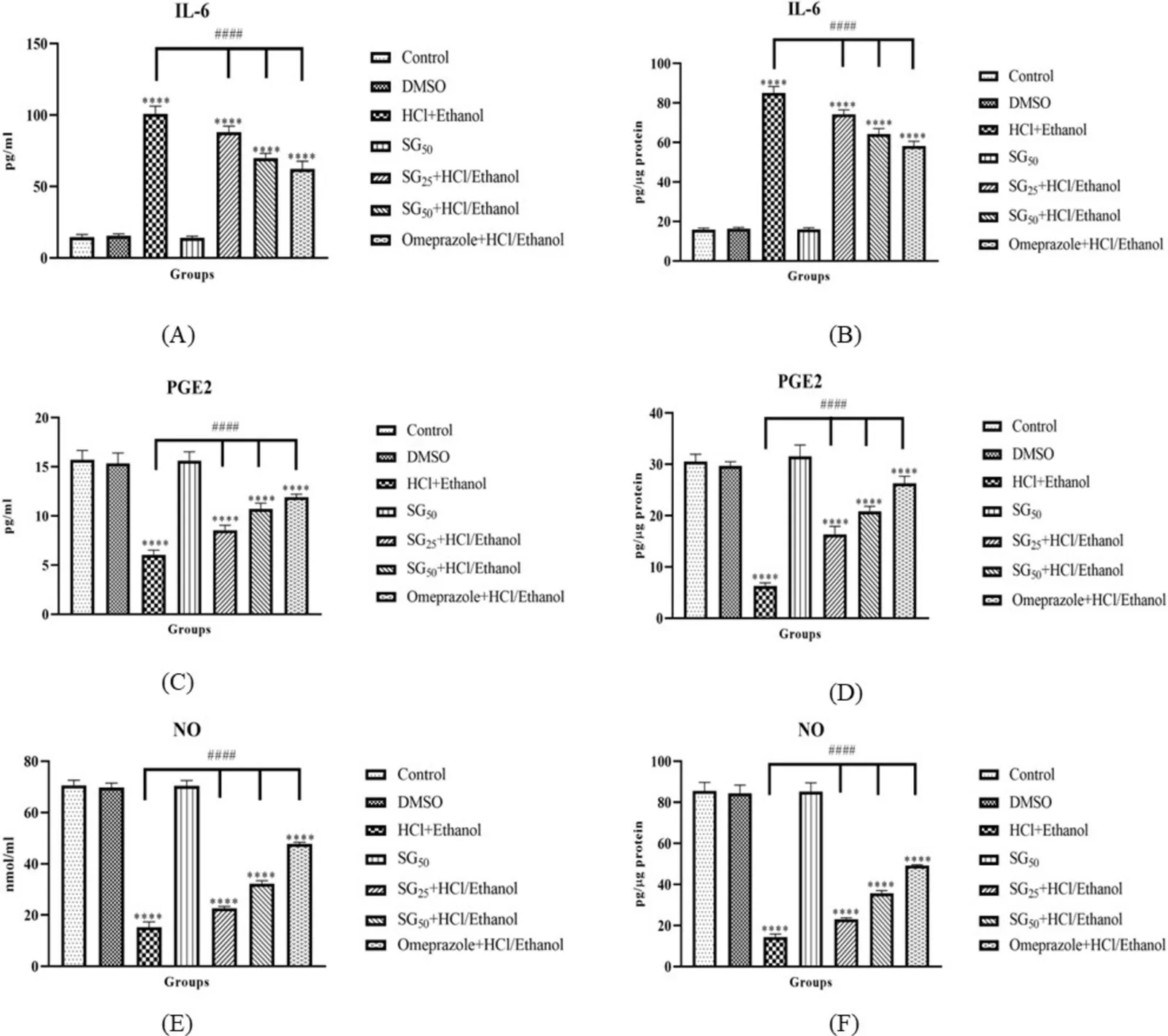
Effects of syringaldehyde (SG) on inflammatory mediators in serum and gastric tissue in ethanol/HCl-induced gastric injury in mice. (**A**) Serum interleukin-6 (IL-6) levels, (**B**) gastric tissue IL-6 levels, (**C**) serum prostaglandin E2 (PGE2) levels, (**D**) gastric tissue PGE2 levels, (**E**) serum nitric oxide (NO) levels, and (**F**) gastric tissue NO levels. Ethanol/HCl administration markedly increased IL-6 levels while significantly decreasing PGE2 and NO levels compared with the control group, indicating pronounced inflammatory and mucosal damage. Pretreatment with syringaldehyde (SG) at doses of 25 and 50 mg/kg significantly attenuated inflammatory responses and restored PGE2 levels in a dose-dependent manner, with effects comparable to omeprazole. Data are expressed as mean ± SD. ****p < 0.0001 vs. Control; ####*p* < 0.0001 vs. HCl/Ethanol group

**Fig. 6:**
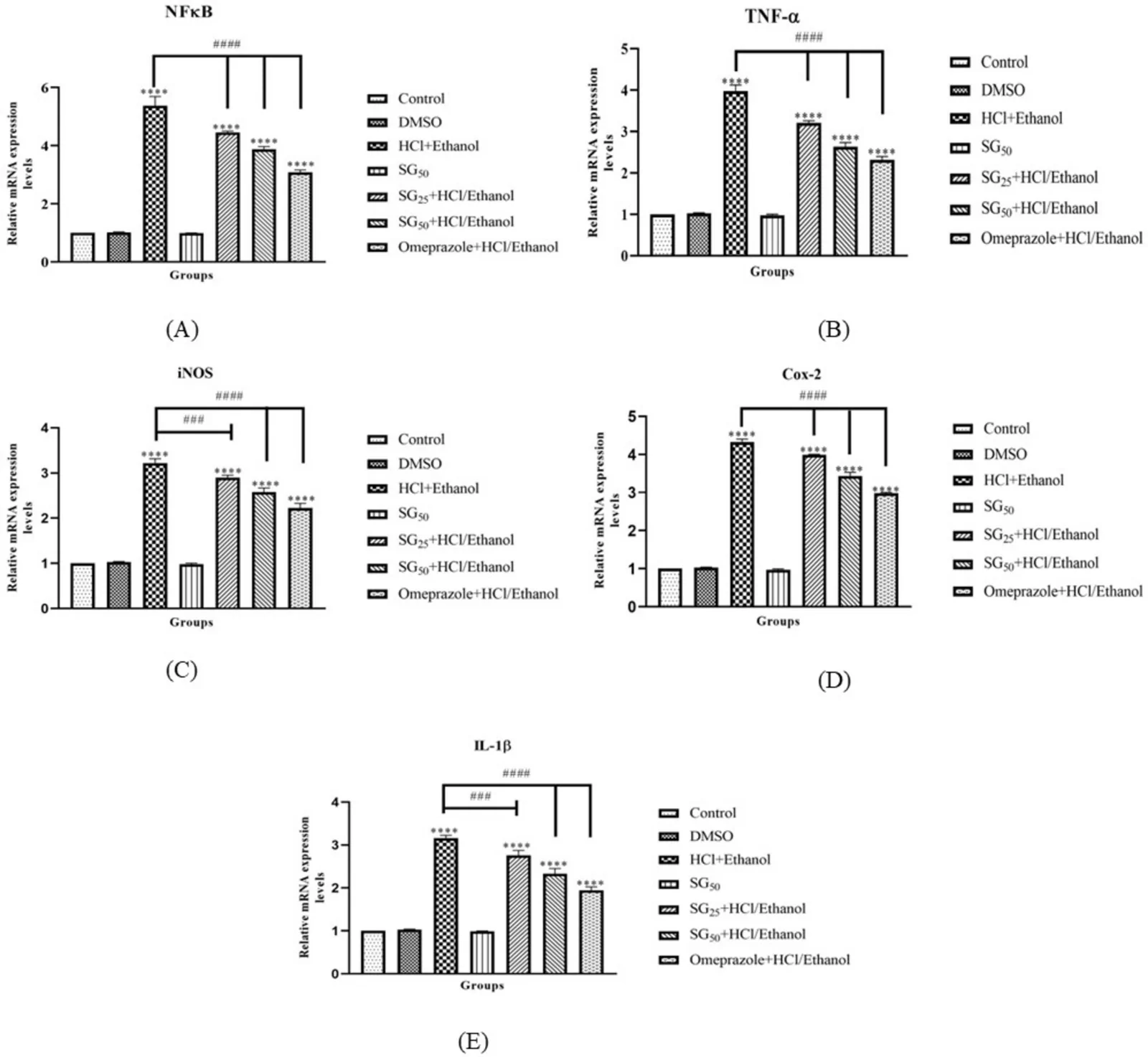
Effects of syringaldehyde (SG) on inflammatory gene expression in gastric tissue of ethanol/HCl-induced gastric injury in mice. Relative mRNA expression levels of (**A**) nuclear factor kappa B (NF-κB), (**B**) tumor necrosis factor-α (TNF-α), (**C**) inducible nitric oxide synthase (iNOS), (**D**) cyclooxygenase-2 (Cox-2), and (**E**) interleukin-1β (IL-1β) in gastric tissue. Ethanol/HCl administration markedly upregulated the expression of pro-inflammatory genes compared with the control group. Pretreatment with syringaldehyde (SG) at doses of 25 and 50 mg/kg significantly downregulated inflammatory gene expression in a dose-dependent manner, comparable to omeprazole. Data are presented as mean ± SD. ****p < 0.0001 vs. Control; ###*p* < 0.001, ####*p* < 0.0001 vs. HCl/Ethanol group

**Fig. 7:**
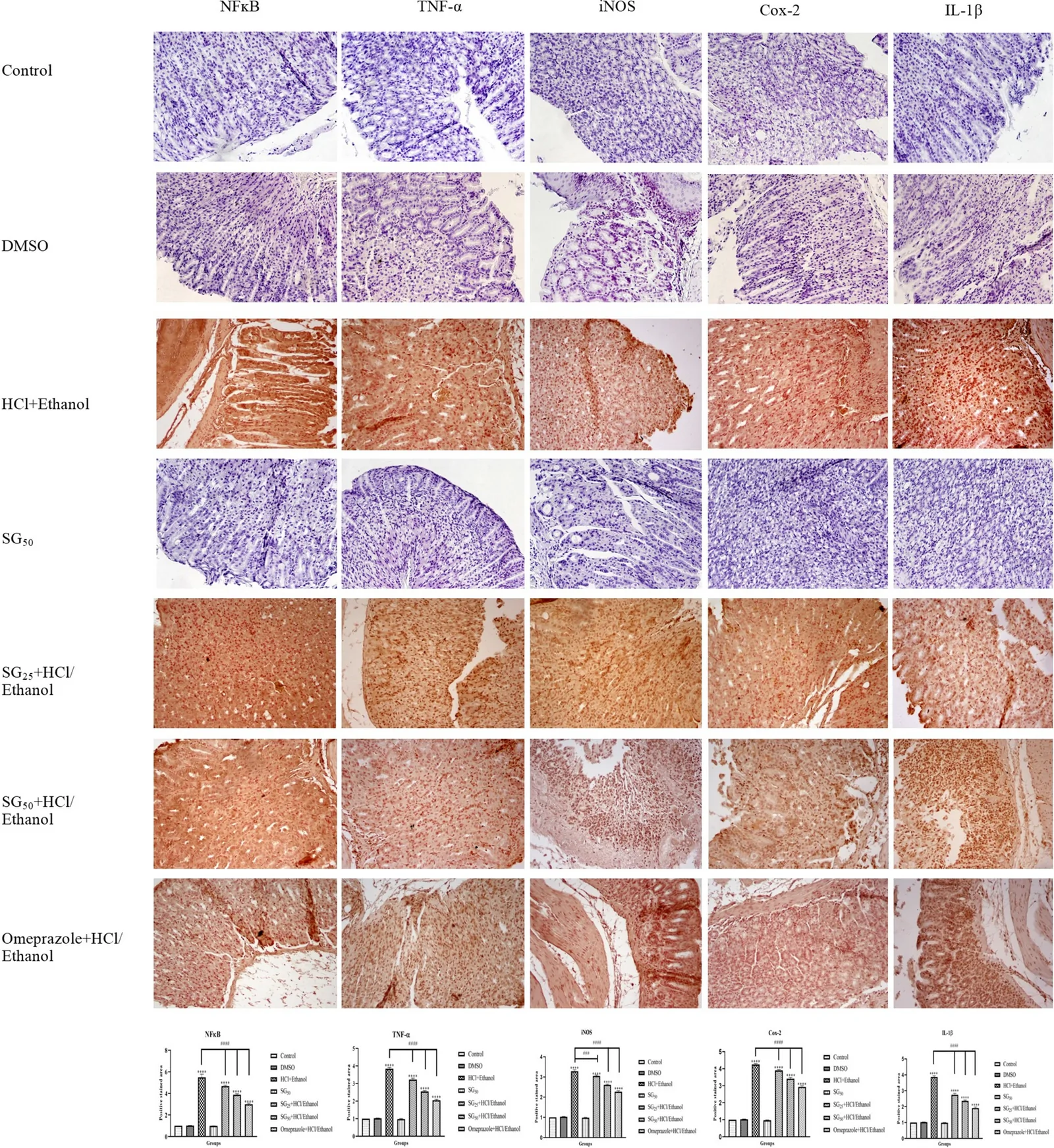
Immunohistochemical evaluation of inflammatory protein expression in gastric tissue of ethanol/HCl-induced gastric injury in mice. Representative immunohistochemical staining images showing the expression of nuclear factor kappa B (NFκB), tumor necrosis factor-α (TNF-α), inducible nitric oxide synthase (iNOS), cyclooxygenase-2 (Cox-2), and interleukin-1β (IL-1β) in gastric tissues from Control, DMSO, HCl/Ethanol, SG₅₀, SG₂₅ + HCl/Ethanol, SG₅₀ + HCl/Ethanol, and Omeprazole + HCl/Ethanol groups. The control and DMSO groups exhibited weak or negligible immunoreactivity. In contrast, the HCl/Ethanol group showed marked increases in immunopositive staining intensity for all inflammatory markers. Pretreatment with syringaldehyde (SG) at doses of 25 and 50 mg/kg significantly reduced immunoreactivity in a dose-dependent manner, comparable to omeprazole treatment. Lower panels present the semi-quantitative analysis of immunopositive staining intensity for each marker. Data are expressed as mean ± SD. ****p < 0.0001 vs. Control; ###*p* < 0.001, ####*p* < 0.0001 vs. HCl/Ethanol group. Original magnification: × 200

Inflammation-related mediators such as NO, PGE2, IL-6, NFκB, TNF-α, iNOS, IL-1β, and Cox-2 play critical roles in the pathogenesis of gastric ulcer. Exposure to HCl/ethanol resulted in a marked increase in IL-6 concentrations in both serum and gastric tissue samples relative to the control group (Fig. 5A–B, respectively), whereas PGE2 (Fig. 5C–D, respectively) and NO (Fig. 5E–F, respectively) levels were markedly reduced (*p* < 0.0001). Moreover, mRNA (Fig. 6A–E) and immunohistochemical (Fig. 7A–E) expression levels of NFκB, iNOS, Cox-2, TNF-α, and IL-1β were significantly increased in the gastric tissues of HCl/ethanol-exposed mice (*p* < 0.0001).

In contrast, treatment with SG at increasing doses and omeprazole significantly regulated the HCl/ethanol-induced alterations in IL-6, PGE2, and NO levels, as well as the mRNA and immunohistochemical expression of NFκB, iNOS, TNF-α, IL-1β, and Cox-2. In addition, no statistically significant differences were observed in the measured parameters in animals treated with DMSO or SG alone (50 mg/kg) relative to the control group.

### Influence of SG on Nrf2/HO-1 signaling and apoptosis-associated markers in gastric tissue following HCl/ethanol exposure

**Fig. 8:**
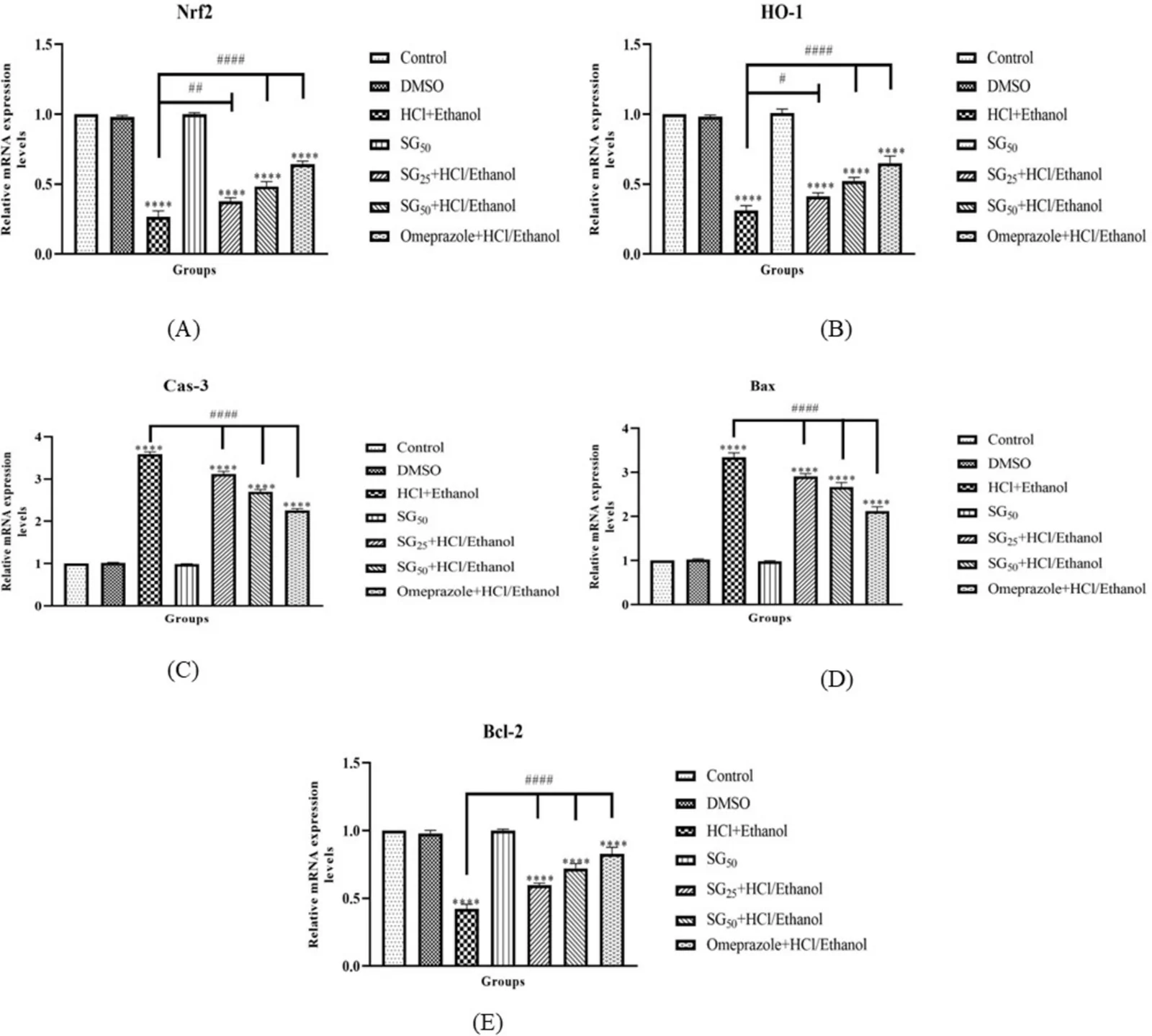
Effects of syringaldehyde (SG) on Nrf2/HO-1 signaling and apoptosis-related gene expression in gastric tissue of ethanol/HCl-induced gastric injury in mice. Relative mRNA expression levels of (**A**) nuclear factor erythroid 2–related factor 2 (Nrf2), (**B**) heme oxygenase-1 (HO-1), (**C**) caspase-3 (Cas-3), (**D**) Bcl-2–associated X protein (Bax), and (**E**) B-cell lymphoma-2 (Bcl-2) in gastric tissue. Ethanol/HCl administration significantly suppressed Nrf2 and HO-1 expression while markedly upregulating pro-apoptotic markers Cas-3 and Bax and downregulating the anti-apoptotic marker Bcl-2 compared with the control group. Pretreatment with syringaldehyde (SG) at doses of 25 and 50 mg/kg significantly restored Nrf2/HO-1 signaling and attenuated apoptosis-related gene expression in a dose-dependent manner, with effects comparable to omeprazole. Data are presented as mean ± SD. ****p < 0.0001 vs. Control; #*p* < 0.05, ##*p* < 0.01, ####*p* < 0.0001 vs. HCl/Ethanol group

**Fig. 9:**
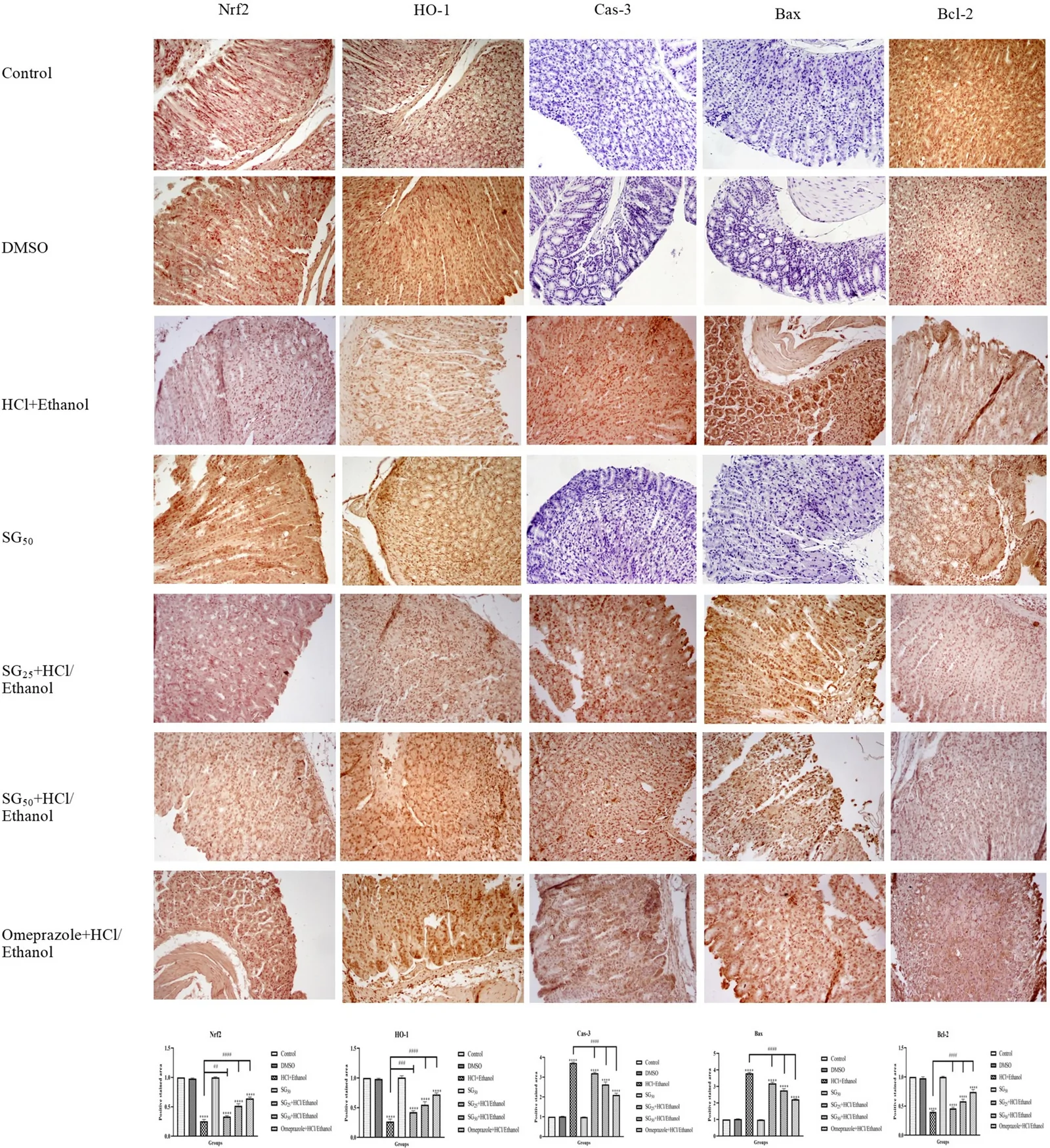
Immunohistochemical evaluation of Nrf2/HO-1 signaling and apoptosis-related protein expression in gastric tissue of ethanol/HCl-induced gastric injury in mice. Representative immunohistochemical staining images showing the expression of nuclear factor erythroid 2–related factor 2 (Nrf2), heme oxygenase-1 (HO-1), caspase-3 (Cas-3), Bcl-2–associated X protein (Bax), and B-cell lymphoma-2 (Bcl-2) in gastric tissues from Control, DMSO, HCl/Ethanol, SG₅₀, SG₂₅ + HCl/Ethanol, SG₅₀ + HCl/Ethanol, and Omeprazole + HCl/Ethanol groups. Control and DMSO groups exhibited strong immunoreactivity for Nrf2, HO-1, and Bcl-2, with weak staining for Cas-3 and Bax. In contrast, the HCl/Ethanol group showed markedly reduced Nrf2 and HO-1 expression along with pronounced increases in Cas-3 and Bax immunoreactivity, indicating enhanced apoptotic activity. Pretreatment with syringaldehyde (SG) at doses of 25 and 50 mg/kg markedly restored Nrf2/HO-1 expression and attenuated Cas-3 and Bax immunoreactivity while enhancing Bcl-2 expression in a dose-dependent manner, comparable to omeprazole treatment. Lower panels present the semi-quantitative analysis of immunopositive staining intensity. Data are expressed as mean ± SD. ****p < 0.0001 vs. Control; ##*p* < 0.01, ###*p* < 0.001, ####*p* < 0.0001 vs. HCl/Ethanol group. Original magnification: × 200

Exposure to HCl/ethanol caused a marked suppression of Nrf2 and HO-1 expression in gastric tissues, as evidenced by both mRNA and immunohistochemical analyses (*p* < 0.0001; Figs. 8A–B and 9). Administration of SG (25 and 50 mg/kg) as well as omeprazole significantly reversed these changes, restoring expression levels toward those observed in control animals. No significant effects were detected in the DMSO or SG-alone (50 mg/kg) groups.

Similarly, HCl/ethanol treatment significantly elevated Cas-3 and Bax expression while reducing Bcl-2 levels at both transcriptional and protein levels (*p* < 0.0001; Figs. 8C–E and 9). These alterations were effectively mitigated by SG and omeprazole treatment, which restored the balance of pro- and anti-apoptotic markers. In contrast, neither DMSO nor SG alone produced any significant changes in these parameters.

## Discussion

Numerous studies have demonstrated that ethanol administration induces acute gastric mucosal injury and is commonly used to establish experimental models of gastric ulcer (Akanda et al. 2018; Li et al. 2021; Xie et al. 2022). The HCl/ethanol-induced gastric ulcer model is a well-established experimental approach for investigating the mechanisms underlying gastric mucosal damage and for evaluating the protective potential of pharmacological agents or natural compounds (Tureyen and Ince 2021; Li et al. 2021). This study evaluated the gastroprotective activity of SG, a phenolic aldehyde reported to possess antioxidant and anti-apoptotic properties (Bozkurt et al. 2014; Shahzad et al. 2020), in a model of HCl/ethanol-induced gastric injury. The results demonstrated that SG markedly alleviated gastric mucosal damage and significantly reduced ulcerative lesions triggered by HCl/ethanol exposure. In addition, SG administration influenced key biological processes involved in gastric injury, including oxidative stress, inflammatory signaling, and apoptosis-related pathways. These protective effects may be partly associated with the modulation of the Nrf2/HO-1 signaling pathway, an important regulator of cellular defense mechanisms against oxidative damage.

The HCl/ethanol mixture induces severe gastric mucosal injury characterized by hemorrhagic lesions and ulceration through mechanisms involving disruption of the mucosal barrier, reduction of mucus secretion, and impairment of mucosal blood flow (Yu et al. 2020). In the present study, pronounced hemorrhage and severe ulcerative lesions were observed in the HCl/ethanol-treated group, findings that are consistent with previous reports (Zhou et al. 2020; Tureyen and Ince 2021). In contrast, administration of SG at increasing doses markedly reduced gastric ulcer severity and was associated with increased gastric pH and enhanced gastric mucus levels. Histopathological evaluation further supported these findings, demonstrating reduced inflammatory cell infiltration and attenuation of submucosal edema. These findings align with earlier reports highlighting the protective roles of natural antioxidant compounds (Akanda et al. 2018; Zhou et al. 2020; Li et al. 2021).

Ethanol-induced gastric damage is closely linked to the overproduction of reactive oxygen species, leading to lipid peroxidation and disruption of cellular redox homeostasis (Qian et al. 2018; Liu et al. 2021). In the current study, exposure to HCl/ethanol led to a pronounced disturbance in oxidative homeostasis, reflected by enhanced lipid peroxidation together with weakened antioxidant defense mechanisms. Administration of SG markedly improved this imbalance, indicating that its protective effect may, at least in part, be attributed to its ability to counteract oxidative stress and support endogenous antioxidant systems (Shahzad et al. 2018, 2020; Tureyen et al. 2025).

PGE2 and NO are key endogenous mediators that play essential roles in maintaining gastric mucosal integrity and promoting ulcer healing (Wallace 2008; Takeuchi 2012; Sánchez-Mendoza et al. 2019). NO exerts gastroprotective effects by regulating mucosal blood flow, enhancing mucus and bicarbonate secretion, and supporting epithelial cell integrity. In parallel, PGE2 contributes to ulcer healing by controlling gastric acid and cytotoxic substance release, stabilizing mast cell membranes, and stimulating tissue repair processes (Wallace 2008; Takeuchi 2012; Zhou et al. 2020). High levels of IL-6 and other inflammatory cytokines are generated as a consequence of ethanol-induced gastric inflammation (Bae et al. 2021). The present study demonstrated that HCl/ethanol exposure markedly reduced gastric PGE2 and NO levels while significantly elevating IL-6 concentrations. In contrast, treatment with omeprazole and SG effectively modulated the ethanol-induced alterations in PGE2, NO, and IL-6 levels. Supporting these findings, previous evidence has shown that SG administration (5–100 µM) to patient-derived peripheral mononuclear cells with myocardial infarction resulted in a pronounced suppression of NO and IL-6 release (Shahzad et al. 2020). Moreover, in mice with experimentally induced arthritis, SG treatment was found to suppress the levels of cytokines such as IL-6 and TNF-α, while concurrently elevating serum concentrations of IL-10 (Li et al. 2023). Overall, these findings suggest that SG may exert gastroprotective effects partly through the modulation of inflammatory mediators and endogenous protective factors such as PGE2 and NO. An apparent discrepancy was observed between increased Cox-2/iNOS expression and reduced PGE2 and NO levels. This may be attributed to severe mucosal damage and oxidative stress induced by HCl/ethanol exposure. Under these conditions, epithelial dysfunction may impair the effective bioavailability of these mediators despite elevated enzyme expression (Sánchez-Mendoza et al. 2019; Zhou et al. 2020).

Exposure to ethanol stimulates immune activity, resulting in increased cytokine production and further amplification of the inflammatory response (Tureyen and Ince 2021). NFκB signaling is a key pathway in the development of gastric inflammation and is linked to the upregulation of inflammatory mediators, including iNOS, IL-1β, TNF-α, and Cox-2 (Omoboyowa et al. 2021). In this study, HCl/ethanol exposure led to a marked increase in the expression of gastric NFκB, Cox-2, IL-1β, iNOS, and TNF-α at both the mRNA and immunohistochemical levels. In contrast, treatment with SG, as well as omeprazole, markedly reduced the expression levels of these inflammatory mediators. In line with our findings, previous studies have reported that SG administration (5–100 µM) to patient-derived peripheral mononuclear cells with myocardial infarction produced a dose-dependent reduction in TNF-α and IL-6 secretion, an effect attributed to inhibition of the NFκB signaling pathway (Shahzad et al. 2020). In models of cardiac injury with isoproterenol, SG treatment (12.5, 25, and 50 mg/kg) was reported to alleviate NO, TNF-α, and IL-6 levels, thereby reducing the production of inflammatory cytokines (Shahzad et al. 2018). Similarly, studies in arthritic mouse models have demonstrated that SG markedly reduces the elevated expression of IL-12p40, IL-23, TNF-α, and IL-6 with these anti-inflammatory effects being mediated by the MAPK/NFκB pathway (Li et al. 2023). Taken together, these findings suggest that SG may exert anti-inflammatory effects in gastric injury partly through modulation of NFκB-related inflammatory signaling. Although SG and omeprazole produced comparable protective effects, their mechanisms of action are likely different. While omeprazole primarily acts through inhibition of gastric acid secretion, the effects of SG may involve modulation of oxidative stress and inflammatory pathways. However, the present findings do not exclude the possibility that SG may exert additional or alternative mechanisms of action, which require further investigation.

The Nrf2 pathway serves as a central cellular defense mechanism against oxidative injury by controlling the transcriptional activation of antioxidant enzymes (Zhou et al. 2020; Ismail et al. 2025; Ali et al. 2025). In addition to its role in antioxidant defense, Nrf2 has been shown to interact with inflammatory signaling pathways, including NFκB and Cox-2, thereby modulating the balance between oxidative stress and inflammation (Kobayashi et al. 2016; Ahmed et al. 2017). Oxidative stress triggers Nrf2 nuclear translocation and upregulates antioxidant enzymes such as HO-1, SOD, and CAT (Xie et al. 2022). In agreement with previous reports (Raish et al. 2021; Yang et al. 2024), this study showed a significant reduction in Nrf2 and HO-1 expression in the gastric tissues of mice subjected to HCl/ethanol exposure. In contrast, treatment with SG, as well as omeprazole, significantly increased Nrf2 expression and was accompanied by elevated HO-1 levels. These findings suggest that modulation of the Nrf2/HO-1 pathway may contribute to SG-mediated protection against HCl/ethanol-induced gastric injury. However, pathway-specific inhibition studies were not performed; therefore, a direct causal relationship cannot be established. Collectively, these findings support the presence of a coordinated regulatory network between oxidative stress and inflammatory mediators rather than a single linear pathway. In agreement with our observations, previous studies have also reported the regulatory influence of SG on the Nrf2/HO-1 pathway (Wen et al. 2024; Tureyen et al. 2025). Oxidative injury resulting from ethanol-induced gastric ulceration promotes activation of the pro-apoptotic protein Bax, triggering the intrinsic apoptotic pathway and ultimately leading to cell death via Cas-3 activation (Zhou et al. 2020). SG administration elevated Bcl-2 levels while decreasing Bax and Cas-3 expression in HCl/ethanol-exposed mice, suggesting that its protective effects may be partly mediated through modulation of apoptosis-related pathways (Bozkurt et al. 2014; Malcok et al. 2020; Tureyen et al. 2025).

**Fig. 10:**
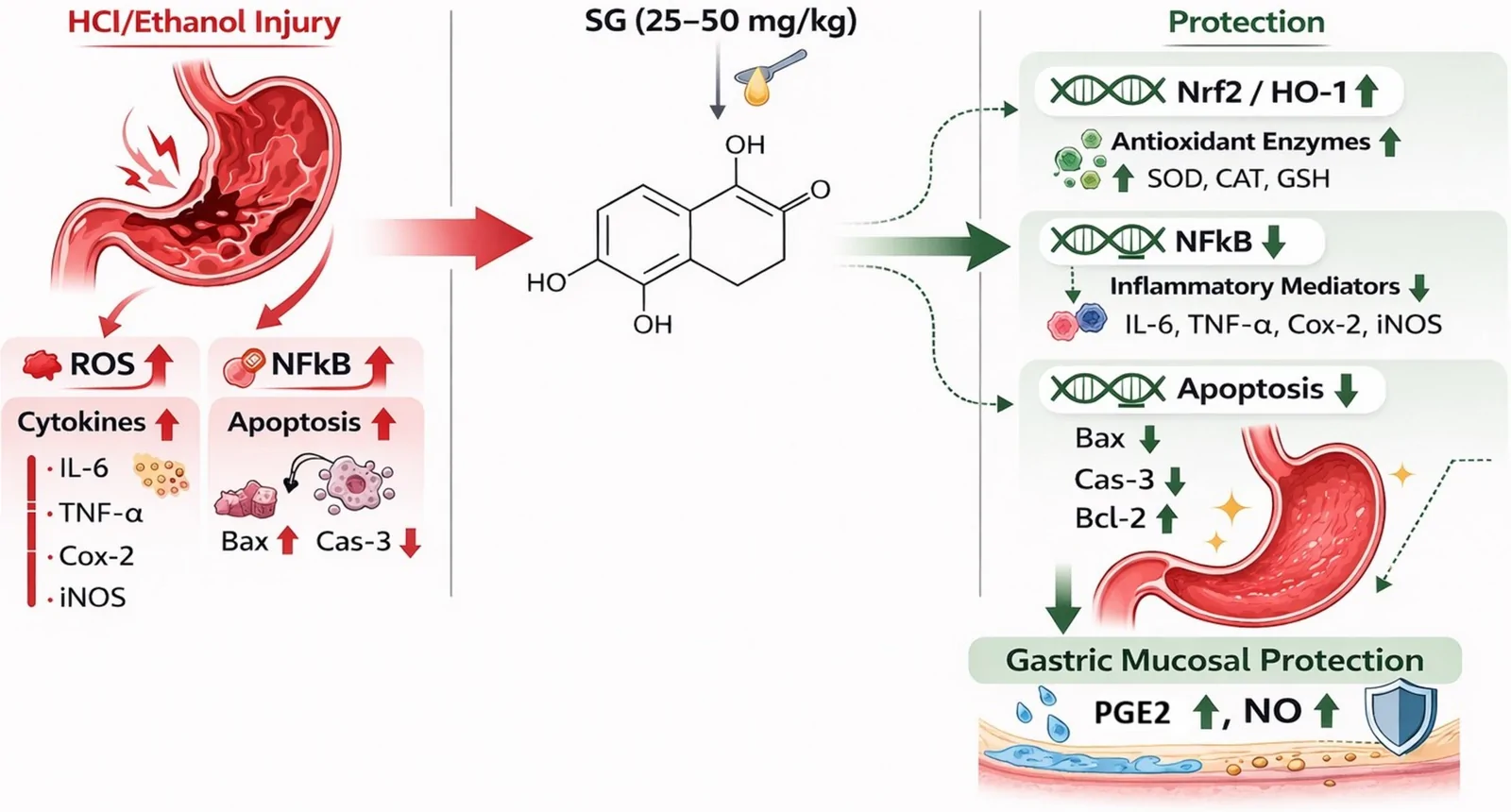
Schematic illustration of the proposed gastroprotective mechanism of syringaldehyde (SG) against HCl/ethanol-induced gastric mucosal injury. Exposure to HCl/ethanol induces oxidative stress, inflammation, and apoptosis, characterized by increased ROS production, activation of NFκB signaling, and upregulation of pro-apoptotic markers. SG may exert its protective effects partly through modulation of the Nrf2/HO-1 pathway, enhancing antioxidant defenses, suppressing NFκB-mediated inflammatory responses, and inhibiting apoptosis. Collectively, these effects contribute to preservation of gastric mucosal integrity through enhancement of PGE2 and NO levels

These findings indicate that SG confers gastroprotective effects, likely associated with its capacity to regulate oxidative stress, inflammation, and apoptosis. The interaction between these processes, along with Nrf2/HO-1 signaling, is illustrated in Fig. 10. The protective action of SG appears to involve strengthening antioxidant defenses, reducing inflammatory activity, and modulating apoptotic pathways. In this context, regulation of the Nrf2/HO-1 axis may represent an important contributing mechanism. To our knowledge, this is the first study to demonstrate the protective potential of syringaldehyde in an HCl/ethanol-induced gastric ulcer model using an integrated biochemical, molecular, and histopathological approach.

Nevertheless, several limitations should be acknowledged. First, the present study did not include loss-of-function experiments targeting the Nrf2 pathway, which would be necessary to establish a causal role of this signaling axis in SG-mediated gastroprotection. Second, apoptosis was evaluated based on gene and protein expression levels of Bax, Bcl-2, and Cas-3, without additional confirmation using cleaved Cas-3 detection or TUNEL staining. Future studies incorporating mitochondrial membrane potential assays may provide additional mechanistic insight into the anti-apoptotic effects of SG. Third, the activation status of NFκB was inferred from expression data rather than nuclear translocation analysis. In addition, western blot validation of major signaling proteins, including Nrf2, HO-1, NFκB p65, and cleaved Cas-3, was not performed and should be considered in future mechanistic investigations.

Fourth, the study lacks in vitro validation using gastric epithelial cell lines (such as GES-1 or RGM-1), which would be important to further confirm the underlying molecular mechanisms. Finally, the HCl/ethanol model represents an acute experimental injury model that does not fully replicate the complexity of chronic human gastric ulcer disease. Additionally, only male mice were used in the present study; therefore, possible sex-dependent differences in gastric ulcer susceptibility, inflammatory responses, and treatment outcomes could not be evaluated.

Despite these limitations, the present findings provide valuable preclinical evidence supporting the protective potential of SG against gastric mucosal injury. Further studies incorporating mechanistic pathway inhibition, detailed apoptosis analyses, and clinical investigations are warranted to clarify the therapeutic applicability of SG in gastric ulcer management.

In summary, the present findings indicate that SG exerts a substantial protective effect against HCl/ethanol-induced gastric damage in mice. This protection appears to be associated with improved redox balance, suppression of inflammatory activity, and regulation of cell death–related mechanisms. The involvement of the Nrf2/HO-1 axis may partially underlie these effects; however, direct mechanistic confirmation is still required. Overall, SG emerges as a potential candidate for gastric mucosal protection, warranting further experimental and clinical validation.

## Data Availability

All source data for this work (or generated in this study) are available upon reasonable request.

## Abbreviations

NSAIDs: Non-steroidal anti-inflammatory drugs
Nrf2: Nuclear factor erythroid 2–related factor 2
HO-1: Heme oxygenase-1
NFκB: Nuclear factor kappa B
TNF-α: Tumor necrosis factor-alpha
Cox-2: Cyclooxygenase-2
SG: Syringaldehyde
DMSO: Dimethyl sulfoxide
HCl: Hydrochloric acid
MDA: Malondialdehyde
GSH: Reduced glutathione
SOD: Superoxide dismutase
CAT: Catalase
NO: Nitric oxide
PGE2: Prostaglandin E2
H₂O₂: Hydrogen peroxide
PBS: Phosphate-buffered saline
iNOS: Inducible nitric oxide synthase
IL-1β: Interleukin-1 beta
Bax: Bcl-2-associated X protein
Cas-3: Caspase-3
Bcl-2: B-cell lymphoma-2

## Declarations

### Artificial intelligence usage statement

Artificial intelligence tools were used solely for language editing purposes. All scientific content, data analysis, and conclusions were produced by the authors.
